# Heparin-binding protein in sepsis: player! predictor! positioning?

**DOI:** 10.1186/s13613-019-0546-3

**Published:** 2019-06-20

**Authors:** Patrick M. Honore, David De Bels, Leonel Barreto Gutierrez, Sebastien Redant, Herbert D. Spapen

**Affiliations:** 1 ICU Department, Centre Hospitalier Universitaire Brugmann-Brugmann University Hospital, Place Van Gehuchtenplein, 4, 1020 Brussels, Belgium; 2 Development, Ageing and Pathology Research Group, Vrije Universiteit Brussel, Brussels, Belgium

In a post hoc study of the multicenter FINNAKI trial, Tverring et al. recently reported that measuring heparin-binding protein (HBP) on admission in the intensive care unit (ICU) improved prediction of sepsis-induced acute kidney injury (AKI). In addition, high plasma HBP levels were associated with a significantly higher fluid balance within 24 h, more organ failure within the first week, and increased 28-day mortality [1]. These observations support HBP as a novel prominent pawn on the already well-stuffed AKI biomarker chessboard.

HBP is stored in azurophilic granules and secretory vesicles of neutrophils and is quickly and abundantly secreted when neutrophils are activated and mobilized. Once released, HBP displays a strong proinflammatory activity. In the blood stream, HBP chemoattracts and activates more neutrophils but also T-lymphocytes and monocytes. Binding of HBP to β2-integrins of monocytic cells initiates intracellular signaling processes which evoke a release of chemokines and proinflammatory mediators [2]. Monocytes roll along, stick to, and finally cross the endothelium. Following extravasation, monocytes differentiate into macrophages that migrate toward the site of infection. HBP contributes to antimicrobial clearance through facilitating recognition and uptake of pathogens by macrophages. In addition, HBP activates endothelial cells and increases endothelial permeability by interacting with luminal cell surface glycosaminoglycans and activating protein kinase C and Rho-kinase pathways [3]. Shedding and destruction of the endothelial glycocalyx exposes adhesion molecules which enhance trapping and transendothelial migration of activated white blood cells. Structural endothelial cell damage associated with a procoagulant state and failing vasomotor tone regulation further disrupt the endothelial barrier function. Subsequent interstitial and tissue edema formation is a key feature in the pathophysiology of sepsis-induced organ failure.

HBP proved to be a valuable diagnostic marker in suspected sepsis [4] and demonstrated good prognostic and discriminatory properties in detecting the most severely ill patients with sepsis [5]. Experimental and clinical evidence supports a prominent role of this protein in the pathophysiology of sepsis-induced organ dysfunction [6]. Tverring et al. now add evidence that HBP measurement may allow early identification of those septic patients at increased risk of AKI.

Development and progression of sepsis-related AKI are determined by endothelial leakage, renal tubular cell inflammation, and an adaptive cell-cycle shutdown effect [7]. These injurious and reparative processes run a complex course, and their mutual interaction is influenced by difficult-to-control variables such as timely diagnosis of sepsis, duration of shock, type and adequacy of resuscitation (the hydroxyethyl starch/sepsis-induced AKI connection!), and impact of therapeutic interventions (e.g., renal replacement therapy). The pathophysiological kinship between HBP, capillary leakage, and renal cell inflammation suggests that HBP may be a useful prognostic marker in patients at risk of sepsis-induced AKI. As such, HBP “rivals” the cell-cycle arrest-based urinary biomarker panel that combines tissue inhibitor of metalloproteinase-2 and insulin-like growth factor-binding protein 7 ([TIMP-2].[IGFBP7]) which is approved and commercialized (NephroCheck™) as an aid to determine whether ICU patients are at risk of developing moderate to severe AKI. The role of the urinary [TIMP-2].[IGFBP7] test to predict sepsis-induced AKI is derived from subgroup analysis of septic patients enrolled in large prospective clinical trials [8]. To date, this predicting ability has not been evaluated prospectively except for an observational study in 98 ICU patients, confirming that [TIMP-2].[IGFBP7] acts as an early predictor of AKI, regardless of sepsis [9]. It must be emphasized that secretion levels of TIMP-2 and IGFBP7 are likely to change over time which may determine test specificity and sensitivity and probably requires adjustment of cutoff values related to the sepsis stage [10].

Targeting HBP modulatory effects in sepsis could prove therapeutically useful. Either preventing HBP receptor binding or HBP release from neutrophils are theoretically attractive options. However, evidence that decreasing HBP plasma levels would improve global or renal outcome is still scarce. Moreover, a specific receptor for HBP has not been identified and unselective blocking of crucial immunologic effects of frontline neutrophils may expose patients to unexpected or unwanted side effects. HBP is highly positively charged and thus can be neutralized by negatively charged molecules such as heparin and colloid solutions. Unfractionated and low molecular weight heparin block HBP-induced endothelial cell permeability [3] and renal tubular cell inflammation [11] in vitro and in a murine model. Suppression of HBP expression by heparin injection following development of AKI in septic mice resulted in a reduction in renal injury severity accompanied by a significantly decreased macrophage infiltration and activation [12]. A meta-analysis including trials investigating heparin administration in patients with sepsis or infection-related disseminated intravascular coagulation revealed a beneficial effect of heparin on mortality. However, heparin-related safety issues (bleeding, transfusion need, and thrombocytopenia) remain insufficiently documented [13]. Albumin is an established colloidal plasma expander in septic shock. Its particular distribution of positive and negative surface charges helps to maintain the integrity of the endothelial glycocalyx [14]. Albumin inhibits HBP-induced endothelial cell permeability but paradoxically increases in vitro renal inflammation [15]. A large clinical trial assessing fluid resuscitation with albumin versus crystalloids in severe sepsis and septic shock did not reveal a difference in AKI incidence [16]. HBP and the [TIMP-2].[IGFBP7] panel both act as marker and player during the septic process. Whether they may serve as beacons for treatment guidance remains to be elucidated. A logical next step would be a prospective head-to-head comparison of plasma HBP and urinary [TIMP-2].[IGFBP7] as AKI predictors in a large cohort of patients undergoing standard and uniform resuscitation for sepsis and septic shock. Such study requires correctly determined biomarker cutoff levels, the preferential use of resuscitation fluids that are least harmful for the kidney (albumin, buffered crystalloids), and upfront therapeutic interventions to combat fluid overload.

## Data Availability

Not applicable.

## Abbreviations

HBP: heparin-binding protein
ICU: intensive care unit
AKI: acute kidney injury
TIMP-2: tissue inhibitor of metalloproteinase-2
IGFBP7: insulin-like growth factor-binding protein 7
